# The Impact of Patient Characteristics on Their Attitudes Toward an Online Patient Portal for Communicating Laboratory Test Results: Real-World Study

**DOI:** 10.2196/25498

**Published:** 2021-12-17

**Authors:** Rosian Tossaint-Schoenmakers, Marise Kasteleyn, Annelijn Goedhart, Anke Versluis, Esther Talboom-Kamp

**Affiliations:** 1 Saltro Diagnostic Centre Utrecht Netherlands; 2 National eHealth Living Lab Leiden University Medical Centre Leiden Netherlands; 3 Public Health and Primary Care Department Leiden University Medical Centre Leiden Netherlands; 4 Unilabs Group Geneva Switzerland

**Keywords:** patient portal, eHealth impact questionnaire, laboratory test results, self-efficacy, usability, age, gender, chronic disease, education, patient characteristics

## Abstract

**Background:**

Patient portals are promising tools to increase patient involvement and allow them to manage their health. To optimally facilitate patients, laboratory test results should be explained in easy language. Patient characteristics affect the usage of portals and the user satisfaction. However, limited research is available, specified for online communicating laboratory test results, on whether portal use and acceptance differ between groups.

**Objective:**

The aim of this study was to assess the effect of patient characteristics (gender, age, education, and chronic disease) on the self-efficacy and perceived usability of an online patient portal that communicates diagnostic test results.

**Methods:**

We used the online-administered eHealth impact questionnaire (eHIQ) to explore patients’ attitudes toward the portal. Patients visiting the portal were asked to complete the questionnaire and to answer questions regarding gender, age, education, and chronic disease. The subscale “information and presentation” of the eHIQ assessed the usability of the patient portal and the subscale “motivation and confidence to act” assessed self-efficacy to determine whether patients were motivated to act on the presented information. Age, gender, education, and chronic disease were the determinants to analyze the effect on usability and self-efficacy. Descriptive analyses were performed to explore patient characteristics, usability, and self-efficacy. Univariable and multivariable regression analyses were performed with age, gender, education, and chronic disease as determinants, and usability and self-efficacy as outcomes.

**Results:**

The questionnaire was completed by 748 respondents, of which 428 (57.2%) were female, 423 (56.6%) were highly educated, and 509 (68%) had no chronic disease. The mean age was 58.5 years (SD 16.4). Higher age, high education, and asthma or chronic obstructive pulmonary disease were significant determinants for decreased usability; respectively, b=-.094, 95% CI -1147 to 0.042 (*P*<.001); b=-2.512, 95% CI -4.791 to -0.232 (*P*=.03); and b=-3.630, 95% CI -6.545 to -0.715 (*P*=.02). High education was also a significant determinant for a lower self-efficacy (b=-3.521, 95% CI -6.469 to -0.572; *P*=.02). Other determinants were not significant.

**Conclusions:**

This study showed that the higher-educated users of a patient portal scored lower on usability and self-efficacy. Usability was also lower for older people and for patients with asthma or chronic obstructive pulmonary disease. The results portal is not tailored for different groups. Further research should investigate which factors from a patient’s perspective are essential to tailor the portal for different groups and how a result portal can be optimally integrated within the daily practice of a doctor.

## Introduction

The involvement of patients is important to allow them to manage their own health. When patients are more engaged, they tend to make better decisions on health behavior [1]. Patient involvement has increasingly been stimulated with digital possibilities [2], such as in patient portals [3,4]. A Dutch patient portal developed by Saltro Diagnostic Center provides patients access to laboratory test results, including explanatory information and visualization [3]. The aim of this portal is to increase patients’ knowledge and to facilitate them to take an active role in their diagnostic process (eg, to ask questions and share opinions to improve the diagnostic process and reduce the risk of diagnostic errors [5]). Patient portals conveying laboratory test results in understandable language can help patients to take a more active role in managing their own health [6]. Therefore, it is recommended to test how patients perceive online portals and test results, for example by using the eHealth impact questionnaire (eHIQ) [7].

In 2019, we investigated patients’ attitudes toward the same portal designed to communicate laboratory test results using the eHIQ [6]. The usability of this portal was rated positively, suggesting that the study participants found the patient portal easy to use, considered it trustworthy and appropriate, and that the provided information was easy to understand. The self-efficacy of the patients received a satisfying score, referring to whether patients were motivated to act on the presented information. It was concluded that the patients were generally positive toward the portal with opportunities to optimize self-efficacy; however, the impact of patient characteristics was not accounted for. Patient characteristics such as gender, age, education, and chronic disease can affect the usage of portals and the user satisfaction [8-10]. Limited research is available, specified for online communicating laboratory test results, on whether portal use and acceptance differ between groups. Further research on potential group differences is necessary to fine-tune the portal, making it acceptable for every user. We aim to replicate the previous study with larger numbers to examine how different groups of patients perceived the portal.

The main aim of this study is to evaluate the effect of gender, age, education, and chronic disease on the usability and self-efficacy of patients using a patient portal designed to communicate laboratory tests.

## Methods

### Design and Participants

A cross-sectional real-world study was conducted between December 2019 and July 2020 to explore the influence of patient characteristics on the usability of a patient portal and on self-efficacy. Patients who viewed their test results in the portal were automatically approached to complete the eHIQ. Age, gender, education level, and chronic disease were measured as well. There were no further inclusion or exclusion criteria.

No personal information was collected, and the data could not be traced back to the individual. Therefore, this study does not fall under the Medical Research Involving Human Subject Act (Wet medisch-wetenschappelijk onderzoek met mensen) and did not require approval from an ethics committee.

### Patient Portal

In 2015, Saltro launched a web-based portal that gives patients access to their own laboratory test results, including understandable explanatory information [3]. The content was created by a team of patients, general practitioners (GPs), communication specialists, and clinical chemists. Researchers estimated the level of health literacy of the information at communication level 1B based on the scales of the Common European Framework of Reference for Languages [11]. Daily, approximately 300 unique individuals look up their laboratory test results with the option to share their results with others.

**Figure 1:**
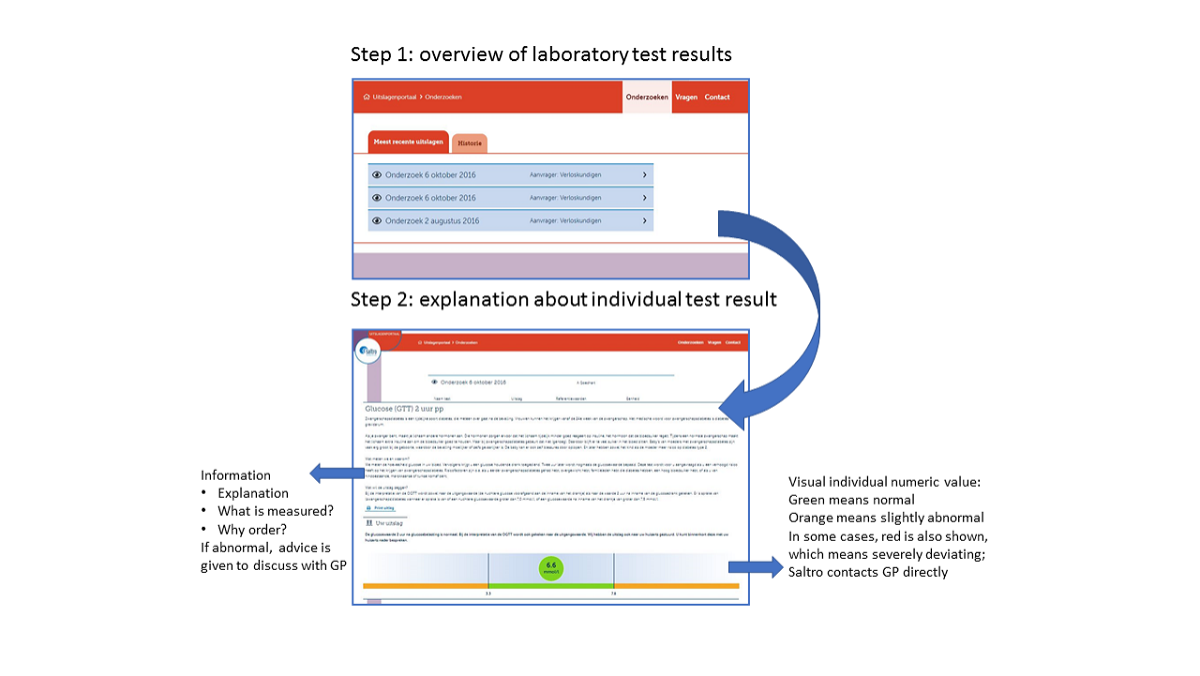
Example of a test result with explanation. GP: general practitioner.

After blood withdrawal, the patients can look up their results by logging into the portal, with a username and password, through the website of the GP. The log-in procedure adheres to Dutch security legislation and guidelines (ie, the Dutch Personal Data Protection Act) and the General Data Protection Regulation guidelines. The patients can see an overview of all laboratory tests ordered by date (Figure 1). Each result has traffic-light–colored bullets and a visual.

This portal can be approached directly for laboratory test results but can also be approached within other portals as a plug-in; for example, a GP portal that functions as medication description.

### Outcome Measures

Primary outcomes were “information and presentation” and “motivation and confidence to act” in the Dutch version of the eHIQ, part 2 (eHIQ2) [12,13]. The eHIQ2 is a self-reporting questionnaire measuring patients’ attitudes toward a specific health-related website. Each of the 26 items is scored on a 5-point Likert scale ranging from “strongly disagree (1)” to “strongly agree (5).” The questionnaire has three subscales: information and presentation; motivation and confidence to act; and identification. The “information and presentation” subscale has 13 items and measures whether people find the website easy to use, which includes items on understanding, trustworthiness, and whether images used were appropriate. This subscale relates to usability. The “motivation and confidence to act” subscale consists of 10 items and assesses whether an individual felt reassured after reading the information on the website and was motivated to manage their health. This subscale relates to self-efficacy. The final subscale, identification, consists of 3 items and measures whether individuals identify with others who use the website. An example item is the following: “I feel I have a sense of solidarity with other people using the website.” As users of the patient portal do not interact with other users, this subscale was considered irrelevant for the current study and is therefore not discussed further. The total scores per subscale were transformed to a 0-100 scale (higher scores representing a more positive attitude).

The determinants were age, gender, education, and chronic disease (Table 1), based on studies demonstrating that portal use was influenced inter alia by age, gender, presence of a chronic illness, education, and health literacy level [8,9,14]. Gender, instead of sex, was chosen because the patients’ attitude and experience were analyzed. There was no biological measurement involved. Education level was chosen, but not health literacy, in order to minimalize the participants’ number of questions. Relationships are proven between health literacy and education level, although health literacy is also common among the highly educated [15]. The choice for types of chronic diseases is based on the 5 most prevalent chronic diseases in the Netherlands: diabetes mellitus, asthma, chronic obstructive pulmonary disease (COPD), cardiovascular disease, and cancer [16,17]. Except for cancer, Saltro performs the blood test for these types of chronic diseases. People with asthma and COPD receive the same pulmonary function test and are therefore considered as 1 patient group in this research. Diabetes mellitus, Asthma or COPD, and cardiovascular diseases are the most prevalent chronic diseases in the population of Dutch GPs; these chronically ill patients are regularly monitored by GPs in a chronic care program with regular laboratory checks.

**Table 1:** Patient characteristics.

Determinant	Variables
Age	Age at completing the questionnaire
**Gender**	
	Male
	Female
**Education** [18]	
	Low (no education, high school)
	Intermediate (intermediate vocational education)
	High (bachelor’s degree, master’s degree, doctorate)
**Chronic disease**	
	Diabetes mellitus
	Asthma or chronic obstructive pulmonary disease
	Cardiovascular disease
	None

### Statistical Analyses

Descriptive analyses were performed to explore patient characteristics, usability, and self-efficacy. Univariable regression analyses were performed with age, gender, education, and chronic disease as determinants and usability and self-efficacy as outcomes. Significant (*P*<.10) determinants were included in multivariable models to examine which characteristics were independently related to the outcomes. To be rather inclusive than exclusive regarding the selection of variables for our multivariable model, *P*=.10 was chosen. A common level of *P*=.05 might fail to include relevant variables in those models [19]. For all other analyses and conclusion, *P*<.05 was considered statistically significant. The analyses were performed using the SPSS version 24 (IBM Corp) [20].

## Results

### Participant Characteristics, Usability, and Self-efficacy

The questionnaire was completed by 748 respondents. Response rate was 1.9% (39,430 unique visitors during the study period). The participants had a mean age of 58.5 years (SD 16.4), and they were mostly female (428/748, 57.2%) and highly educated (423/748, 56.6%) (Table 2). Moreover, 509/748 (68%) had no chronic disease. The mean scores of usability and self-efficacy were 68.9 (SD 10.6) and 62.5 (SD 13.1), respectively (Table 3). The mean (SD) scores on all items of the “information and presentation” and “motivation and confidence to act” domains can be found in Multimedia Appendix 1.

**Table 2:** Patient characteristics (N=748).

Characteristics		Values
Age (years), mean (SD)		52.8 (16.4)
**Gender, n (%)**		
	Male	314 (42.0)
	Female	428 (57.2)
	Missing value	6 (0.8)
**Education, n (%)**		
	Low (no education, high school)	96 (12.8)
	Intermediate (intermediate vocational education)	220 (29.4)
	High (bachelor’s degree, master’s degree, doctorate)	423 (56.6)
	Missing value	9 (1.2)
**Chronic disease, n (%)**		
	Diabetes mellitus	93 (12.4)
	Asthma or COPD^a^	54 (7.2)
	Cardiovascular disease	87 (11.6)
	No chronic disease	509 (68.0)
	Missing value	5 (0.7)

^a^COPD: chronic obstructive pulmonary disease.

**Table 3:** Mean scores on the eHealth impact questionnaire (eHIQ); N=747.

Subscale	Value
Usability^a^, mean (SD) (1 missing^b^)	68.9 (10.6)
Self-efficacy^c^, mean (SD) (1 missing)	62.5 (13.6)

^a^Usability is measured with the eHIQ subscale "information and presentation".

^b^Missing value: one respondent gave the same answer to every question, including reversed questions, which indicates false responding.

^c^Self-efficacy is measured with the eHIQ2 subscale “motivation and confidence to act.”

### Determinants for Perceived Usability

Age, education level, and chronic disease were relevant determinants with *P*<.10 for usability in the univariable analysis; they and where subsequently added in the multivariable model (Table 4). Multivariable analysis showed that higher age and high education were associated with a decreased usability: respectively, *b*=-.094, 95% CI -1147 to -0.042 (*P*<.001); and *b=*-2.512, 95% CI -4.791 to -0.232 (*P*=.03). Chronic disease affected usability, with patients with asthma or COPD scoring significantly lower compared with those without a chronic disease (*b*=-3.630, 95% CI -6.545 to -0.715; *P*=.02).

**Table 4:** Determinants for perceived usability.

Determinant			Univariable analysis			Multivariable analysis	
Reference group	Determinant	*b*^a^ (95% CI)		*P* value	*b* (95% CI)		*P* value
Age per year	Age	-.067 (-0.114 to -0.021)		.004	-.094 (-1.147 to -0.042)		<.001
Male	Gender	1.322 (-0.234 to 2.878)		.10	-.153 (-1.806 to 1.500)		.86
Low education	Intermediate education	1.275 (-1.224 to 3.774)		.32	1.262 (-1.189 to 3.712)		.31
	High education	-1.992 (-4.302 to 0.318)		.09	-2.512 (-4.791 to -0.232)		.03
No chronic disease	Diabetes	-.377 (-2.692 to 1.939)		.80	.347 (-2.053 to 2.747)		.78
	Asthma or COPD^b^	-3.399 (-6.337 to -.416)		.02	-3.630 (-6.545 to -0.715)		.02
	Cardiovascular disease	-.286 (-2.668 to 2.096)		.81	.890 (-1.576 to 3.357)		.48

^a^*b*: unstandardized beta value.

^b^CODP: chronic obstructive pulmonary disease.

### Determinants for Perceived Self-efficacy

Education level was a relevant determinant for self-efficacy in the univariable analysis with *P*<.10 (*b*=-3.521, 95% CI -6.469 to -.572; *P*=.02) (Table 5). Other determinants were not relevant; therefore, there was no need for a multivariable analysis.

**Table 5:** Determinants to perceived self-efficacy.

Determinant			Univariable analysis	
Reference group	Determinant	*b*^a^ (95% CI)		*P* value
Age per year	Age	-.035 (-.095 to 0.024)		.24
Male	Gender	-1.490 (-3.478 to 0.498)		.14
Low education	Intermediate education	.159 (-3.031 to 3.348)		.92
	High education	-3.521 (-6.469 to -0.572)		.02
No chronic disease	Diabetes	-1.279 (-4.254 to 1.697)		.40
	Asthma or COPD^b^	-2.438 (-6.214 to 1.338)		.21
	Cardiovascular disease	2.205 (-.856 to 5.265)		.16

^a^*b*: unstandardized beta value.

^b^CODP: chronic obstructive pulmonary disease.

## Discussion

### Principal Findings

This study aimed to evaluate the impact of patient characteristics on the perceived usability and self-efficacy of a patient portal. Higher education was associated with decreased usability and self-efficacy. Furthermore, usability was lower for older patients and for patients with asthma or COPD. The eHIQ is a validated questionnaire, and the results of this study with the eHIQ are in line with our previous study [6].

The finding that highly educated people have a significantly lower perceived usability and self-efficacy after using the portal is not in line with other research projects [21]. Mostly, people with high education tend to be more eHealth literate, showing more positive outcomes (motivation, self-efficacy, and better interaction with the doctor) after reading health information on the internet [21]. The use of qualitative interviews with the participants to explore the usability findings would be worthwhile. Nonetheless, other research projects on digital health information showed that tailoring—enabling users to self-tailor the preferred mode of information delivery via text and (audio)visuals—enhanced satisfaction with attractiveness and comprehensibility as compared with various versions of the nontailored digital information [22,23]. The patients were directly involved in the design phase of the studied results portal. However, the portal is not tailored for a specific group and might not be suitable for highly educated people. The continued development of the portal is an opportunity to take into account, especially by involving different education groups to give tailored advice through the portal.

This study also revealed that older participants scored lower on the usability of the portal. In other studies, the differences between age groups could be explained via the groups' digital skills. Van Deursen et al [24] and Broekhuizen et al [25] found that a higher age lowered operational and formal internet skills, such as operating an internet browser and maintaining a sense of orientation. However, in a study about the association of the usage of a public evidence-based health website and health care consultations, the use of digital information led to a decrease in regular doctors’ consultations for older people in the same way as for other age groups [26]. Nevertheless, the presentation and design of test results should be tailored for every age group [27] to obtain excellent usability and self-efficacy.

Furthermore, our research demonstrated that patients with asthma or COPD were more negative about usability. Other research projects reported that these patients are often insufficiently capable of understanding health information [28], which could be explained by anxiety, specific illness perception, age, and disease severity [29]. Other studies showed that the use of COPD self-management platforms is higher when the platform is an integrated part of health care [4]. Finally, some studies emphasized the importance of integrating skill-building activities into comprehensive education programs that enable patients with severe cases of asthma or COPD to identify high-quality sources of web-based health information [30]. Our study revealed that asthma or COPD patients are more negative about the results portal. Even more important for this group is tailoring the portal and integrating it into usual care [4]. Therefore, considerations for redesigning the online portal are at issue, together with COPD patients.

### Strength and Limitations

A strength of our study was the high sample size and that the patients completed the questionnaire immediately after they viewed their results, thereby limiting recall bias and giving an accurate picture of the patients’ attitudes toward the portal. Nevertheless, those who completed the study questionnaire were a small portion of the total group that used the patient portal. The low response rate precludes generalizing whether the patient portal display and explanation of the results are acceptable and informative for all of the patients. In future research, it is interesting to compare patients that use the portal to those who do not.* *Moreover, we were not exhaustive with the possible patient characteristics as determinants. We cannot determine other factors that contribute to the patients' perceived usability and self-efficacy after seeing their lab results online. Possible other determinants that may impact usability and self-efficacy are the quality of the portal, the motivation to use the internet for health improvement [31], and the way patients use their knowledge in relation to the doctor [32,33]. Regarding the patient portal itself, lab results need to be easily understandable [34], and technology needs to be easy to use [8,35]. Previous research shows that the related lab results are easily understandable and that the patient portal is easy to use [3]. Therefore, it is interesting to explore which other factors influence a patient's attitude toward the patient portal.

### Conclusions

Highly educated users of a test results portal scored lower on usability and self-efficacy. The usability was also lower for older people and for patients with asthma or COPD. Result portals must adapt the language and communication used, according to the different target groups of age, education, and chronic illness. Only then can users take full advantage of the online information provision. Further research is necessary to determine promoting factors that users themselves consider important in a results portal, in order to tailor it for different groups. Further research is also needed on ways in which a portal can be optimally implemented and integrated within the daily practice of a doctor.

## Appendix

Multimedia Appendix 1 Mean (SD) score subscales “information and presentation” and “motivation and confidence to act” from the eHealth impact questionnaire (eHIQ).

## Abbreviations

COPD: chronic obstructive pulmonary disease
eHIQ: eHealth impact questionnaire
GP: general practitioner
