# Supplementary material for: The Impact of Patient Characteristics on Their Attitudes Toward an Online Patient Portal for Communicating Laboratory Test Results: Real-World Study
Source: JMIR Form Res. 2021 Dec 17;5(12):e25498. doi: 10.2196/25498 (PMC8726048; doi:10.2196/25498)
Supplement: Multimedia Appendix 1 [file formative_v5i12e25498_app1.docx]

**Appendix. Mean (SD) scores subscales “Information and presentation” and “Motivation and confidence to act” (eHIQ).**

**Table 1.** Means and standard deviations (SDs) of the two subscales of the eHIQ-part 2 and the individual items.

| Subscale | Item | Mean (SD) |
| --- | --- | --- |
| **Information and presentation** | |  |
|  | I trust the information on the website | 4.1 (0.6) |
|  | I can easily understand the information on the website | 4.2 (0.7) |
|  | The language on the website made it easy to understand | 4.1 (0.7) |
|  | The information on the website left me feeling confused^a^ | 4.1 (0.8) |
|  | I value the advice given on the website | 3.8 (0.7) |
|  | The website is easy to use | 3.9 (0.7) |
|  | The website provides a wide range of information | 3.7 (0.7) |
|  | The website has a positive outlook | 3.7 (0.8) |
|  | The people who have contributed to the website understand what is important to me | 3.6 (0.7) |
|  | On the whole, I find the website reassuring | 3.6 (0.7) |
|  | I found the images on the website distressing^a^ | 3.5 (0.7) |
|  | The website includes useful tips on how to make life better | 3.4 (0.7) |
|  | Photographs and other images were used appropriately on the website | 3.3 (0.6) |
| **Motivation and confidence to act** | |  |
|  | The website helps me to have a better understanding of my personal health | 3.8 (0.7) |
|  | The website encourages me to take actions that could be beneficial to my health | 3.8 (0.9) |
|  | The website gives me confidence that I am able to manage my health | 3.6 (0.8) |
|  | The website encourages me to play a more active role in my healthcare | 3.5 (0.8) |
|  | I have learnt something new from the website | 3.4 (0.9) |
|  | I feel more inclined to look after myself after visiting the website | 3.4 (0.8) |
|  | The website prepares me for what might happen to my health | 3.5 (0.8) |
|  | I would consult the website if I had to make a decision about my health | 3.4 (0.8) |
|  | The website makes me more confident to discuss my health with the people around me (for example, my family, or people at work) | 3.3 (0.9) |
|  | The website gives me the confidence to explain my health concerns to others | 3.3 (0.8) |

**Note.** eHIQ = eHealth Impact Questionnaire.

^a^ This item was reversed scored.
